# Integrating Target and Shadow Features for SAR Target Recognition

**DOI:** 10.3390/s23198031

**Published:** 2023-09-22

**Authors:** Zhiyuan Zhao, Xiaorong Xue, Iqra Mariam, Xing Zhou

**Affiliations:** School of Electronics and Information Engineering, Liaoning University of Technology, Jinzhou 121001, China

**Keywords:** synthetic aperture radar (SAR), SAR image classification, features of target and shadow, convolutional neural network (CNN), attention mechanism

## Abstract

Synthetic aperture radar (SAR) sensor often produces a shadow in pairs with the target due to its slant-viewing imaging. As a result, shadows in SAR images can provide critical discriminative features for classifiers, such as target contours and relative positions. However, shadows possess unique properties that differ from targets, such as low intensity and sensitivity to depression angles, making it challenging to extract depth features from shadows directly using convolutional neural networks (CNN). In this paper, we propose a new SAR image-classification framework to utilize target and shadow information comprehensively. First, we design a SAR image segmentation method to extract target regions and shadow masks. Second, based on SAR projection geometry, we propose a data-augmentation method to compensate for the geometric distortion of shadows due to differences in depression angles. Finally, we introduce a feature-enhancement module (FEM) based on depthwise separable convolution (DSC) and convolutional block attention module (CBAM), enabling deep networks to fuse target and shadow features adaptively. The experimental results on the Moving and Stationary Target Acquisition and Recognition (MSTAR) dataset show that when only using target and shadow information, the published deep-learning models can still achieve state-of-the-art performance after embedding the FEM.

## 1. Introduction

Synthetic aperture radar (SAR), an active imaging sensor, can operate under all-day and all-weather conditions and deliver high-resolution images [1]. SAR has extensive applications in various civilian and military domains, such as geological surveying, climate change monitoring, and environmental surveillance [2]. Despite the wealth of data generated by SAR, manually extracting relevant information is impractical; hence, automatic target recognition (ATR) has become a crucial aspect of SAR image interpretation.

SAR ATR is generally divided into three steps: detection, discrimination, and classification [3]. The classification stage can be further divided into feature extraction and classifier design. Feature extraction reduces the dimensionality of the raw SAR images and extracts highly discriminative features from the raw input for classifiers to perform classification tasks. Standard classifiers in the SAR ATR field include support vector machines (SVM) [4,5,6], sparse representation classifier (SRC) [7,8], and multilayer perceptron (MLP) [9]. In recent years, researchers have designed various methods to extract different features from SAR images, which can be categorized into three types: handcrafted features, depth features, and fusion features.

Handcrafted features are mainly designed for the unique characteristics of SAR images, including geometric structure features, transform domain features, and scattering features. For example, the moment features describe the geometric structure information of the target and shadow regions, such as area, center, centroid, and [10,11,12,13]. In addition, descriptors encode or extract features from the contours of the target and shadow, using techniques such as Fourier descriptors, elliptic Fourier descriptors, and Zernike moments [4,12,14,15]. Fourier transform, Wavelet transform, Gabor transform, and principal component analysis (PCA) could be used to extract the features from SAR images [9,16]. Scattering features in SAR ATR mainly involve attributed scattering centers (ASCs) [17]. SAR ATR employing scattering features typically relies on template matching or region matching methods, which define a similarity measure between these features and assign the target label to the template class with the highest similarity [18,19,20,21]. Although the handcrafted features designed for the target and shadow in SAR images have physical explainability about geometric information or the scattering mechanism, the overall ATR performance is not outstanding. The reason for this is that a kind of feature cannot describe in-depth information about the target or shadow; however, combining multiple features may fail to provide robust feature representation due to redundancy or high correlation between different features.

Depth features are extracted by convolutional neural networks (CNN). Recently, CNN-based methods have achieved extraordinary recognition accuracy in the field of SAR image classification [22,23,24,25,26,27]. Profeta et al. [22] developed AFRLeNet, a network specifically designed for the seven-classification problem of SAR images. To address the issue of overfitting in deep neural networks for SAR image classification, Chen et al. [27] proposed a fully convolutional neural network called A-ConvNets. Furthermore, with the advancements in computer vision, attention mechanisms have been introduced in SAR image target recognition. For instance, Zhan et al. [28] proposed the AM-CNN combined with the CBAM, which achieved a classification accuracy of 99.35% on a 10-class MSTAR dataset. Lang et al. [29] integrated a multidomain attention module into CNN, which fused features from the frequency domain and the wavelet transform domain to enhance the model’s feature extraction capability. Park et al. [30] proposed a novel channel attention DS-AE, based on the squeeze-and-excitation (SE)mechanism, to preserve the integrity of model channel information. Although depth feature-based ATR models demonstrate outstanding classification accuracy, the mapping relationship between the model’s input and output is challenging to interpret intuitively. Moreover, mainstream CNN models typically take the original SAR image as input. This makes it difficult for them to extract helpful depth information from shadows due to the unique properties of shadows.

Fusion features can use the complementarity between different features to improve ATR performance further. Examples include the fusion of Gabor features and depth features in [31], and the combination of Gabor features and texture features in [32]. In [31], Gabor features and depth features are combined by initializing the inception blocks in the Inception network with multi-scale and multi-directional Gabor filters. Additionally, the combination of depth features and other handcrafted features also has achieved good recognition results, such as the combination of depth features with gradient features [33], depth features with transform domain features [34,35], and depth features with texture features [36]. Currently, the fusion of depth and scattering features is also gaining attention. On the one hand, data-driven depth features provide highly discriminative features for classification. On the other hand, ASC features based on scattering theory provide physical interpretability that depth features do not have. The effective combination of both has spawned a wealth of research on SAR ATR [37,38,39,40,41].

**Figure 1:**
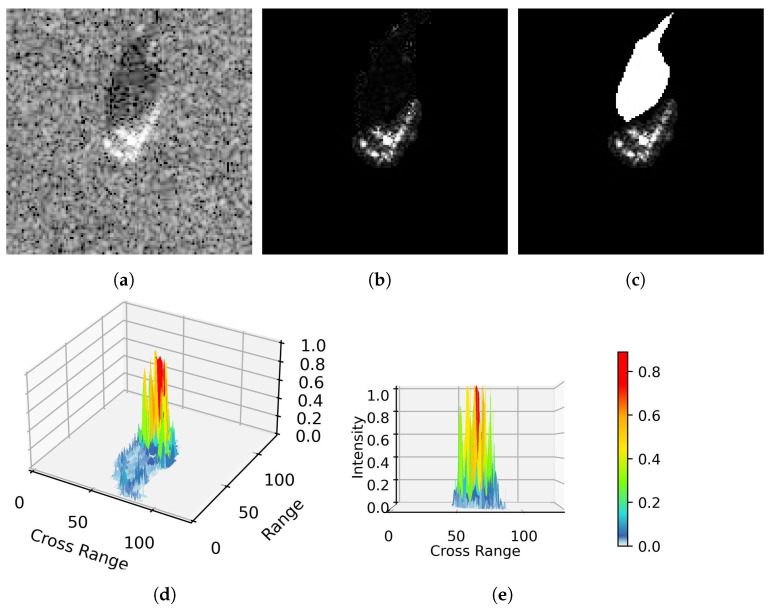
Target and shadow intensity distribution in SAR image. (**a**) Original SAR image. (**b**) Target and shadow region. (**c**) Target region and shadow mask. (**d**) 3D view of target and shadow region. (**e**) Side view.

Fusion features have become prevalent in SAR ATR, research on the fusion of shadow and depth features has not been explored in depth. SAR sensors operate under the condition of slant-viewing, which produces shadow regions in the resulting SAR image. Shadows can indirectly represent the targets, such as their outlines and heights. Considering this, traditional methods focus on extracting geometric properties or contour information from shadows [7,13,14,15]. Although these methods have computational advantages, they struggle to capture deep representations of shadows. It is possible to use CNN as a feature extractor to fuse depth information of shadows and targets for classification automatically. However, existing CNN-based SAR ATR methods often directly employ the original image as input, which suppresses the expression of shadow features. There are two possible reasons for this situation. Shadows have low amplitude, and they are sensitive to the depression angle. These two unique attributes make it difficult for CNN to utilize shadow features effectively. First, the formation of the shadow is due to the occlusion of the high object, causing an area of the scene not to produce radar echoes [42]. Therefore, the intensity of the shadow is much lower than the target one, see Figure 1d,e. If the target and shadow regions are directly fed to CNN without processing, it will harm extraction of targets depth features [43]. Second, it is difficult for the shadow to provide a stable representation of targets due to its high sensitivity to the radar’s depression angle. According to our current understanding, current research on the combination of shadows and deep CNN networks is not in depth. Choi et al. [44] proposed a dual-branch CNN structure to separately extract depth features from the preprocessed target region and shadow region. However, this ignores the relative position relationship between the target and shadow. The relative position of the target and shadow reflects the radar viewing angle and target attitude during imaging, which can provide helpful discriminative information for the classifier [13].

Therefore, to enable CNN to utilize depth features of both targets and shadows comprehensively, the contributions of this paper are as follows.

(1) We first propose a segmentation method based on statistical features of the SAR image to extract regions of targets and shadows. This preprocessing allows us to compensate for the unique attributes of shadows to help the CNN extract the depth information of shadows. Then, we use the target region and shadow mask as input of the CNN, which not only solves the low-intensity problem of the shadow but also restricts the CNN to extract depth features from shadow contours, see Figure 1c.

(2) A data-augmentation method is proposed to provide a robust representation of shadows. Based on the shadow imaging geometry, this method can not only compensate for the geometric distortion caused by different imaging depression angles but also increase the diversity of the training set to prevent overfitting.

(3) We propose a novel feature-enhancement module (FEM) based on DSC and CBAM. The attention-based FEM can comprehensively extract high-discriminative features of target regions and shadow masks. Specifically, we introduce a spatial attention mechanism in the FEM, allowing it to fuse the depth features of targets and shadows adaptively. We also perform interpretability analysis on FEM and spatial attention in FEM to further explore its enhancement effect.

The rest of this paper is organized as follows. In Section 2, we first introduce the SAR image segmentation method, followed by the data-augmentation method and the specific details of FEM. Experiments and analysis based on the MSTAR dataset are in Section 3. Finally, Section 4 provides conclusion.

## 2. Methodology

**Figure 2:**
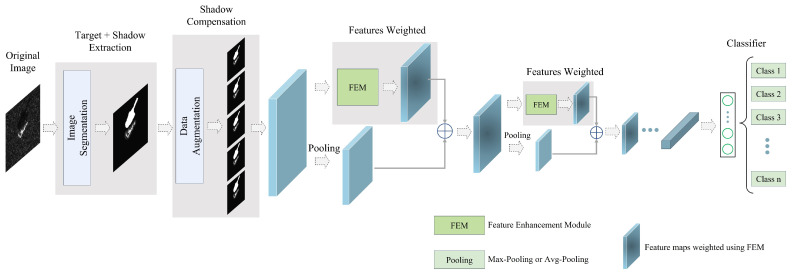
The proposed framework of SAR target recognition based on the target region and shadow mask. The FEM is embedded into the downsampling layer of CNN models and weights the feature maps of targets and shadows.

The overall framework proposed in this paper is shown in Figure 2. This framework includes three main modules. First, SAR images are segmented to extract target areas and shadow masks. Then, data augmentation is applied to the segmentation results to increase the diversity of training samples and compensate for the geometric distortion of shadows. Finally, the proposed FEM is embedded into existing deep CNN models for feature extraction and classification. Each module is explained in detail below.

### 2.1. SAR Image Segmentation

A simple SAR image scene typically consists of three components: the target area, the shadow area, and the background clutter. The intensity distributions of the target and shadow regions exhibit different characteristics, as illustrated in Figure 1. Therefore, a simple threshold-based method, relying on statistical models, can be employed to separate the target and shadow regions from the SAR image [13,23,44]. Although threshold-based segmentation effectively extracts target regions, it may not be entirely suitable for shadow extraction due to the influence of speckle noise in SAR images and the occlusion caused by other objects. Filtering methods commonly used in optical images, such as median filtering and Gaussian filtering, are not appropriate for mitigating non-additive speckle noise in SAR images [14]. Therefore, anisotropic diffusion filtering has been introduced for denoising SAR images, as discussed in [14,15]. Anisotropic diffusion filtering can effectively suppress SAR image noise while preserving the structural information of the target and shadow regions. Motivated by [15,44,45], we propose a method for extracting the shadow mask based on the target centroid-labeled. This method first employs anisotropic diffusion filtering to denoise the SAR image, followed by a dual thresholding approach to roughly segment the target and shadow regions. Finally, the Euclidean distance between the centroid of the target contour and the centroids of suspicious shadow contours is used to filter out false shadows, therefore enhancing the robustness of shadow segmentation.

This paper first extracts the target mask based on the method in [44]. Then, the centroid of the target mask is used as auxiliary information to extract the shadow mask. Suppose an original SAR image is represented as I(x,y), where 1≤x≤M and 1≤y≤N, of size M×N. The following is the detailed process of segmentation.

*Step 1:* Apply a logarithmic transformation to I(x,y) to enhance low grayscale value regions, resulting in $I_{log}$.

*Step 2:* Perform anisotropic diffusion filtering on $I_{log}$ to obtain $I_{pm}$.

*Step 3:* Normalize $I_{pm}$ to obtain $I_{n}$, where $I_{n}=I_{pm}/\mathrm{sum}\left(I_{pm}\right)$.

*Step 4:* Binarize $I_{n}$ by marking positions with intensities above 3% as 1 and the rest as 0, resulting in the target mask $T_{b}$. Similarly, mark positions with intensities below 6% as 1 and the rest as 0, obtaining the shadow mask $S_{b}$.

*Step 5:* Apply a sliding window of size W×W to perform counting filter processing on $T_{b}$ and $S_{b}$ separately, yielding the counting filter results $T_{c}$ and $S_{c}$.

*Step 6:* Perform morphological dilation and closing operations on $T_{c}$ and $S_{c}$ separately.

*Step 7:* Select the largest connected region as the final target mask $T_{mask}$, and compute its centroid $\left(t_{x},t_{y}\right)$.

*Step 8:* Calculate the centroids of binary regions in $S_{c}$ and the Euclidean distances *d* between each centroid and $\left(t_{x},t_{y}\right)$. Select the largest connected region with $d<T_{D}$ as the final shadow mask $S_{mask}$.

*Step 9:* Obtain the target region and shadow mask image by applying $T_{mask}\times I\left(x,y\right)+S_{mask}$.

**Figure 3:**
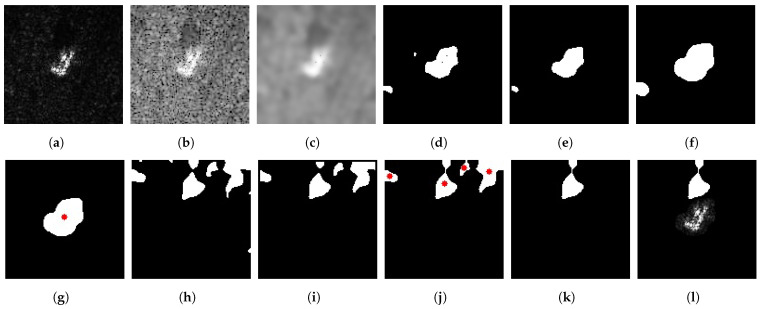
The stepwise output of the proposed segmentation method on a SAR image. (**a**) Original ZSU23/4 image. (**b**) ZSU23/4 image in the log scale. (**c**) Image after denoising. (**d**) Target mask after upper threshold processing (3%). (**e**) Target mask after count filtering. (**f**) Target mask by morphological dilation. (**g**) The largest connected region is the target mask; the red dot indicates its centroid. (**h**) Shadow mask after lower threshold processing (6%). (**i**) Shadow mask after count filtering. (**j**) Suspicious shadow masks after morphological closing; red dots indicate their centroids. (**k**) Refined shadow mask processed using distance threshold ($T_{D}$). (**l**) Final target region and shadow mask.

The parameter details of the proposed SAR image segmentation algorithm are in Section 3.2. To provide a more intuitive understanding of each step in the algorithm, Figure 3 presents the stepwise output of the segmentation. As seen in Figure 3c, applying anisotropic diffusion filtering to the SAR image not only helps suppress speckle noise but also preserves the structural and detailed information of the target and shadow regions, resulting in smooth contours of the segmented shadow masks.

### 2.2. Data Augmentation

Given the characteristics of SAR images where shadows do not directly reflect the high backscattering of targets, the intensity of shadow areas tends to be relatively low or even close to 0, as depicted in Figure 1. Therefore, shadows can only provide auxiliary information about targets, such as their contours. Some traditional SAR ATR methods leverage this characteristic by extracting geometric features from the binarized shadow mask (contour) instead of directly extracting features from the shadow area. For instance, geometric properties such as a shadow mask’s center, centroid, and moment features can be extracted [10,11,12,13]. Alternatively, descriptors can be employed to encode the shadow contours directly, enabling the extraction of contour features [13,14,15]. Motivated by these approaches, we propose to combine the shadow mask with a target region as input for the deep-learning model. This circumvents the problem of significant intensity differences between the shadow and the target and guides subsequent deep networks to extract features from the shadow contours. Moreover, this processing method preserves the relative positional relationship between the target and the shadow.

**Figure 4:**
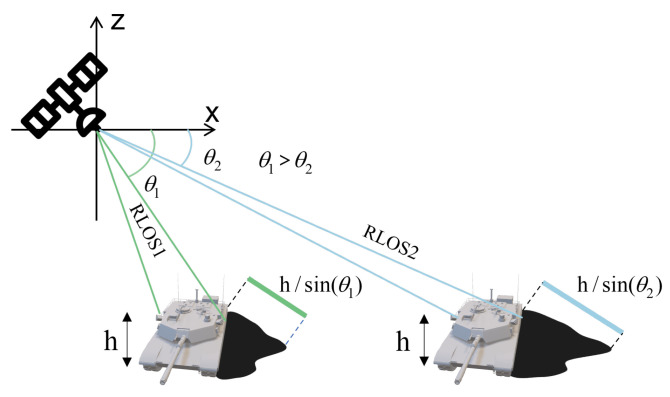
SAR projection geometry under different depression angles.

However, shadows tend to exhibit unstable characteristics due to their sensitivity to depression angles in SAR images. Geometric distortions occur in both the target and shadow areas of SAR images at different radar depression angles. These distortions lead to variations in the shape and position of targets and shadows in training and test data, posing challenges for SAR target recognition. Figure 4 illustrates the projection of ground objects under different radar line of sight (RLOS) conditions. As depicted in Figure 4, the projections of the target and shadow areas in the range direction experience compression with scaling factors of cos(θ) and 1/sin(θ), respectively, where θ represents the depression angle of the radar [44,46]. For example, the SOC training and test set images under MSTAR (as described in Section 3.1) are generated at depression angles of 17° and 15°, respectively. Consequently, the scaling factor for the target region is:

(1) $$\lambda_{\mathrm{target}\;}=\frac{\cos\left(\theta_{\mathrm{test}\;}\right)}{\cos\left(\theta_{\mathrm{train}\;}\right)}=\frac{\cos\left(15^{\circ }\right)}{\cos\left(17^{\circ }\right)}\approx 1.01$$

where $\lambda_{\mathrm{target}\;}$ denotes the scaling factor of the target region. However, the scaling factor of the shadow is larger than the target area, namely:

(2) $$\lambda_{\mathrm{shadow}\;}=\frac{1/\sin\left(\theta_{\mathrm{test}\;}\right)}{1/\sin\left(\theta_{\mathrm{train}\;}\right)}=\frac{\sin17^{\circ }}{\sin15^{\circ }}\approx 1.13$$

where $\lambda_{\mathrm{shadow}\;}$ is the scaling factor of the shadow region. Due to the scaled characteristic of targets and shadows, we use affine transformation to geometrically adjust the image in the training set to compensate for the geometric distortion of the training set compared to the test set. Take the affine transformation of the shadow as an example. Assuming that the shadow mask in the Cartesian coordinate system is S[x,y], after applying the affine transformation, it becomes $S[x^{\prime },y^{\prime }]$, and its coordinate mapping can be calculated as follows [44,46]:

(3) $$\left(\begin{array}{c} x^{\prime }\\ y^{\prime } \end{array}\right)=\left(\begin{array}{cc} \lambda_{\mathrm{shadow}\;} & 0\\ 0 & 1 \end{array}\right)\left(\begin{array}{c} x\\ y \end{array}\right).$$

**Figure 5:**
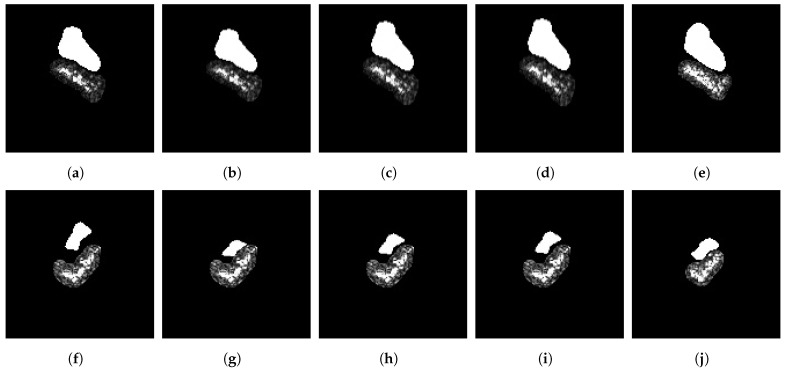
Example of performing data augmentation on 2S1 and BRDM2. (**a**) 2S1 image at azimuth angle $100^{\circ }$ on training set. (**b**–**d**) 2S1 images enhanced with scale factors 0.95, 1.05, and 1.15, respectively. (**e**) 2S1 image at azimuth angle $100^{\circ }$ on test set. (**f**) BRDM2 at azimuth angle $35^{\circ }$ on training set. (**g**–**i**) BRDM2 images enhanced with scale factors 0.58, 0.73, and 0.88, respectively. (**j**) BRDM2 image at azimuth angle $35^{\circ }$ on test set.

Considering that the images in the MSTAR dataset are collected at azimuth angles ranging from 0° to 360° with intervals of 5° to 6°, there may be some deviation in the scaling factor. To address this, we applied four scaling parameters, namely [0.95,1.15], with a step size of 0.05 to the shadow mask. As a result, the newly generated training set is five times larger than the original. Figure 5 illustrates the augmented images obtained by applying different scaling factors to the 2S1 and BRDM2 images in the training set. It is important to note that though the scaling factor for the target area is small, we simultaneously performed an affine transformation on both the target and shadow to preserve their relative positional relationship. This data-augmentation technique not only increases the diversity of training samples to prevent overfitting in the deep-learning model but also compensates for the geometric distortion in the target and shadow areas caused by different depression angles during imaging. Thus, the augmented training set becomes more representative of the data distribution in the test set.

### 2.3. Feature-Enhancement Module

The low intensity and instability of shadows can be solved by binarized masking and data augmentation, respectively, but the importance of targets and shadows is different. In other words, the target region contains rich scattering information, while the shadow mask can only provide the indirect expression of the target. Moreover, compared to the original image, the CNN only takes the target region and shadow mask as input, which significantly reduces the available information during deep feature extraction, especially when the pooling layers compress the spatial resolution and cause more severe information loss.

Considering the above issues, we propose a feature-enhancement module (FEM) based on DSC and CBAM. First, the CBAM in FEM adaptively fuses essential features of the target and shadow for classification. Second, the module has enough generalization capability so that we do not need to change existing backbone networks and can directly embed FEM into their downsampling layers. Finally, and not least importantly, it can enhance the feature extraction capability of the deep-learning model and compensate for the loss of features after pooling. This section first introduces DSC and CBAM. Detailed information about FEM is then provided.

#### 2.3.1. Depthwise Separable Convolution

The MobileNets series has recently gained popularity for their ability to achieve high accuracy in image classification while being lightweight enough to run on mobile and embedded devices [47,48,49]. A key innovation in these networks is the introduction of depthwise separable convolution (DSC).

**Figure 6:**
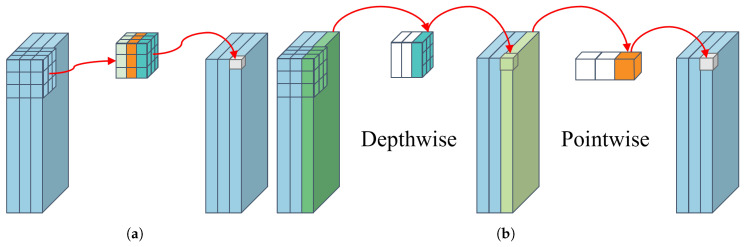
Comparison of standard convolution and depthwise separable convolution (DSC). (**a**) Standard convolution. (**b**) Depthwise separable convolution.

DSC differs from standard convolution by decomposing it into two separate steps: depthwise convolution and pointwise convolution. In standard convolution, computations are performed simultaneously in spatial and channel dimensions. However, DSC performs these computations in two distinct stages. First, depthwise convolution executes convolution operations on each channel of the input feature map individually. Then, pointwise convolution linearly combines the results of the depthwise convolution using 1×1 convolutions [47]. By decomposing the convolution in this way, DSC significantly reduces the number of trainable parameters in the CNN. Figure 6 illustrates the differences between standard convolution and DSC.

Assuming the application of a standard convolution with kernel $K\in R^{k\times k\times M\times N}$ to the input feature map $X\in R^{D_{x}\times D_{x}\times M}$, resulting in feature map $Y\in R^{D_{y}\times D_{y}\times N}$, where k represents the spatial size of the convolution kernel $K,D_{x}$ and $D_{y}$ are the heights and widths of the input and output feature maps, respectively, and M and N denote the number of channels in the input and output feature maps, respectively,

(4) $$Y_{k,l,n}=\sum_{i,j,m}K_{i,j,m,n}\cdot X_{k+i-1,l+j-1,m}.$$

The computation of DSC is divided into two processes, namely depthwise convolution and 1×1 convolution. The depthwise convolution kernel $\hat{K}\in R^{k\times k\times M}$ is used for channel-wise filtering of the feature map, i.e., the m-th filter of $\hat{K}$ is convolved with the m-th channel of X,

(5) $${\hat{Y}}_{k,l,m}=\sum_{i,j}{\hat{K}}_{i,j,m}\cdot X_{k+i-1,l+j-1,m}.$$

A pointwise convolution is then performed on the result of the depthwise convolution. Finally, the reduction of the DSC compared to the standard convolution can be calculated as:

(6) $$\begin{array}{c} R=\frac{k\cdot k\cdot M\cdot D_{x}\cdot D_{x}+M\cdot N\cdot D_{x}\cdot D_{x}}{k\cdot k\cdot M\cdot N\cdot D_{x}\cdot D_{x}}=\frac{1}{N}+\frac{1}{k^{2}}. \end{array}$$

As seen from (6), DSC can significantly reduce the computational cost of the model compared to standard convolution. Considering that the number of the output feature map channels N is usually large, the computational expense of using a 3×3 DSC is approximately 1/9 that of standard convolution.

#### 2.3.2. CBAM

**Figure 7:**
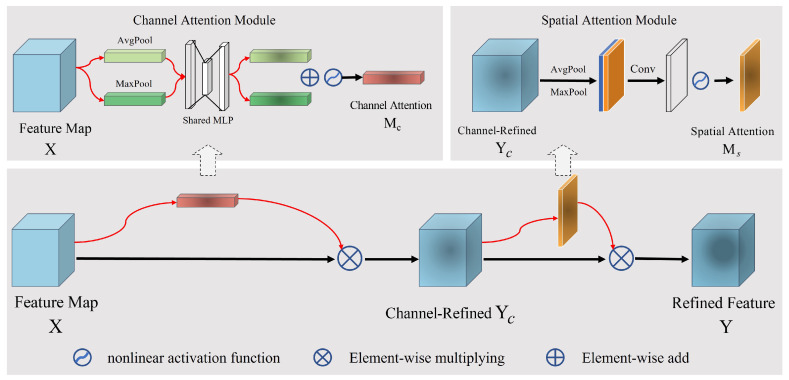
The network topology of CBAM. The upper left is the channel attention module, and the upper right is the spatial attention module.

The convolutional block attention module (CBAM) is an attention mechanism that can adaptively adjust the weights of different spatial positions and channels in the feature map to improve the performance of the model [50]. The CBAM module consists of channel and spatial attention, as shown in Figure 7. Given a feature map $X\in R^{H\times W\times C}$, CBAM first infers the attention weights $M_{c}\in R^{1\times 1\times C}$ in the channel dimension and then infers the attention weights $M_{s}\in R^{H\times W\times 1}$ in the spatial position, where *H*, *W* and *C* represent the height, width and the number of channels of the feature map, respectively. The calculation process is as follows [50]:

(7) $$\begin{array}{cc} \; & Y_{c}=M_{c}\left(X\right)⊗X,\\ \; & Y=M_{s}\left(X\right)⊗Y_{c}, \end{array}$$

where ⊗ represents element-wise multiplication, and Y is the refined output of X after passing through the CBAM. The specific calculation method of the channel attention weight $M_{c}$ is:

(8) $$\begin{array}{cc} M_{c}\left(X\right) & =\sigma (\mathrm{MLP}(\mathrm{AvgPool}(X))+\mathrm{MLP}(\mathrm{MaxPool}(X)))\\ \; & =\sigma \left(W_{1}\left(W_{0}\left(X_{\mathrm{avg}}^{c}\right)\right)+W_{1}\left(W_{0}\left(X_{\max}^{c}\right)\right)\right) \end{array}$$

where σ represents the nonlinear activation function, MLP denotes a multilayer perceptron with weights $W_{0}\in R^{C/r\times C}$ and $W_{1}\in R^{C\times C/r}$, r is the reduction ratio and $X_{\mathrm{avg}}^{c}\in R^{1\times 1\times C}$ and $X_{\max}^{c}\in R^{1\times 1\times C}$ represent the average pooling and max pooling results of X in the spatial dimension, respectively. As can be seen from the channel attention module in Figure 7, the computation of channel attention first applies global average pooling and global max pooling on the spatial dimension of feature map X to generate average-pooled feature $X_{\mathrm{avg}}^{c}$ and max-pooled feature $X_{\max}^{c}$, used to describe spatial context information. Then, a shared fully connected layer is used to weight the average-pooled and max-pooled features further. As a result, the channel attention mechanism can adaptively adjust the weight of each channel, enhancing the representation of valuable features and reducing noise interference from irrelevant features.

The computation of spatial attention is similar to channel attention. However, it performs global average pooling and global max pooling on the feature map X in the channel dimension to obtain average-pooled feature $X_{\mathrm{avg}}^{s}\in R^{H\times W\times 1}$ and max-pooled feature $X_{\max}^{s}\in R^{H\times W\times 1}$, respectively. Then, the two are concatenated along the channel dimension and passed through a standard convolution to obtain a 2D spatial attention weight. That is:

(9) $$\begin{array}{cc} M_{s}\left(X\right) & =\sigma (\mathrm{Conv}([\mathrm{AvgPool}(X);\mathrm{MaxPool}(X)]))\\ \; & =\sigma \left(\mathrm{Conv}\left(\left[X_{\mathrm{avg}}^{s};X_{\max}^{s}\right]\right)\right) \end{array}$$

where σ represents the nonlinear activation function, and Conv denotes a standard convolution operation. As shown in Figure 7, spatial attention focuses on which positions in the feature map have richer information. In other words, it adaptively weights different spatial positions of feature maps of the targets and shadows to emphasize the most useful features for classification.

#### 2.3.3. Feature-Enhancement Module

**Figure 8:**
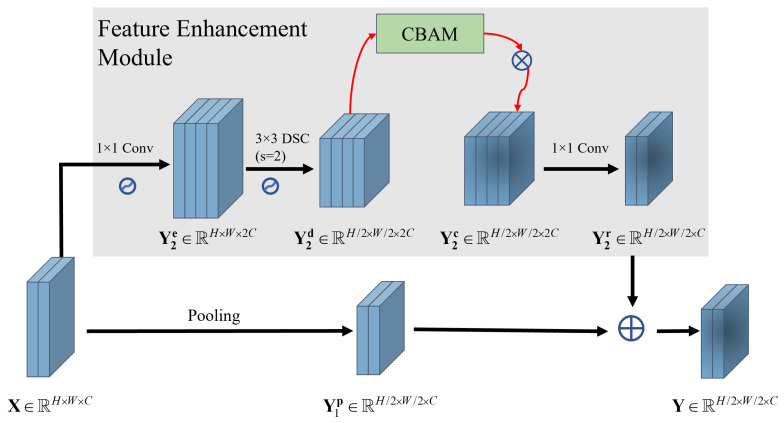
The network topology of the proposed FEM. FEM is embedded in the downsampling layer of the CNN.

The FEM primarily comprises the inverse residual block and CBAM, as illustrated in Figure 8. The inverse residual block utilizes DSC to expand the input feature map in the channel dimension and downsample it in the spatial dimension [49]. CBAM then assigns distinct weights to different spatial positions and channels of the feature map, emphasizing the spatial and channel importance of the feature map of target and shadow, respectively [50].

To provide more detail, given a feature map $X\in R^{H\times W\times C}$, the pooling operation first downsamples X to obtain $Y_{1}^{p}\in R^{H/2\times W/2\times C}$. Subsequently, the convolution kernel $K_{1}\in R^{1\times 1\times C\times 2C}$ is used to expand the channel dimension of the input feature map X, producing a new feature map $Y_{2}^{e}\in R^{H\times W\times 2C}$. Then, depthwise convolution (see (5)) is applied for further feature extraction and downsampling, resulting in $Y_{2}^{d}\in R^{H/2\times W/2\times 2C}$. Using Equation (7), the spatial and channel dimensions of $Y_{2}^{d}$ are weighted to generate the CBAM-refined feature map $Y_{2}^{c}\in R^{H/2\times W/2\times 2C}$. The convolution kernel $K_{2}\in R^{1\times 1\times 2C\times C}$ is then convolved with $Y_{2}^{c}$ to acquire the final enhanced feature map $Y_{2}^{r}\in R^{H/2\times W/2\times C}$. Lastly, residual connections are used to connect the pooled feature map $Y_{1}^{p}$ and the enhanced feature map $Y_{2}^{r}$:

(10) $$Y=Y_{1}^{p}+Y_{2}^{r}.$$

**Figure 9:**
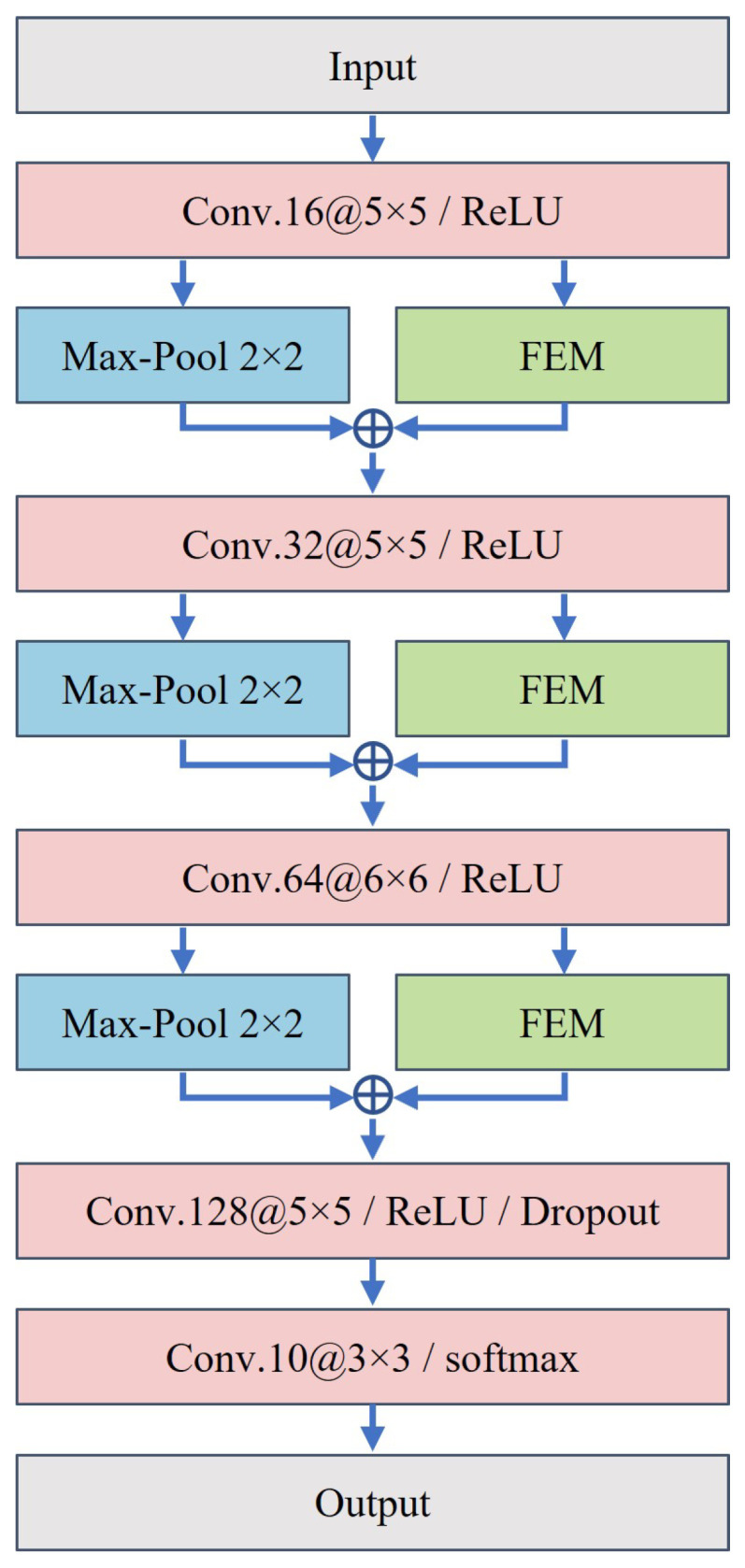
The network structure of A-ConvNets after embedding FEM.

Here, Y represents the enhanced feature map. The FEM employs the inverse residual block based on DSC, which is lightweight and does not significantly increase the number of trainable parameters of the original models. Furthermore, by integrating spatial and channel attention within CBAM, the FEM adaptively can fuse the depth representation of the target region and the shadow mask, prioritizing the most relevant parts for classification. For example, Figure 9 displays the detailed network structure of A-ConvNets [27] with the added FEM.

## 3. Experiments and Analysis

In this section, we design a series of experiments to validate the gains of the proposed shadow mask and FEM under different conditions and analyze the interpretability of the FEM. We first present the dataset used in this paper and different operating conditions. Next, under different experiment configurations, we incorporate FEM into several existing deep network models to calculate its gains. The final part is the interpretability analysis of FEM.

### 3.1. Dataset

**Figure 10:**
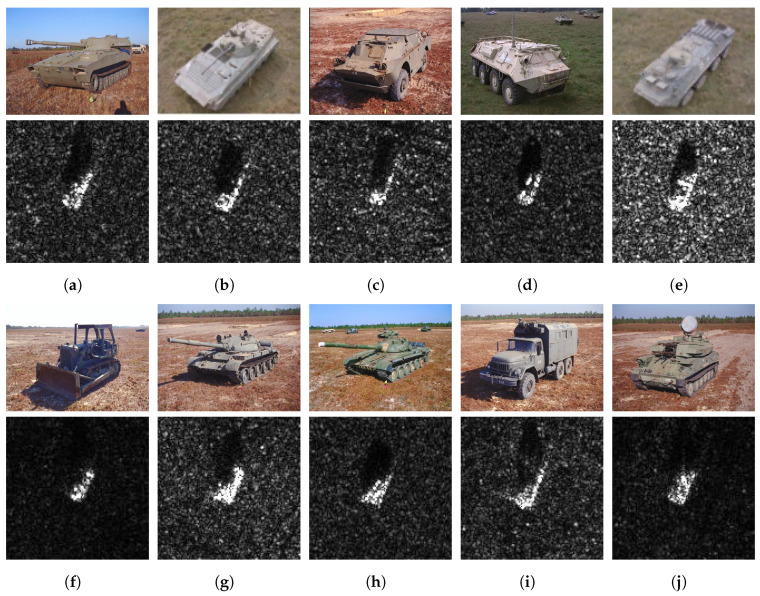
Optical and SAR images of ten different types of ground targets. (**a**) 2S1. (**b**) BMP2. (**c**) BRDM2. (**d**) BTR60. (**e**) BTR70. (**f**) D7. (**g**) T62. (**h**) T72. (**i**) ZIL131. (**j**) ZSU23/4.

The Moving and Stationary Target Acquisition and Recognition (MSTAR) dataset is generated by collecting high-resolution spotlight SAR images of former Soviet ground target military vehicles under different imaging conditions [27]. SAR sensors acquire target slices at every 5° to 6° in all azimuth viewing angles. The MSTAR includes ten different ground target types (rocket launcher: 2S1; armored personnel carrier: BMP2, BRDM2, BTR60, BTR70; bulldozer: D7; tank: T62, T72; truck: ZIL131; air defense unit: ZSU23/4). The spatial resolution of each class is 0.3 m × 0.3 m, with image sizes of nearly 128×128 pixels. Figure 10 shows the optical images of these targets and their corresponding SAR images.

As different imaging conditions can cause changes in the distribution of SAR image data, the MSTAR dataset typically includes two types of data sets to evaluate the algorithm’s generalization: standard operating conditions (SOC) and extended operating conditions (EOCs) [51]. In SOC, images of the training and test sets were obtained under similar depression angles (17° for training and 15° for testing). Table 1 shows detailed information on the ten target types under SOC.

In contrast, in EOCs, the training and test sets differ significantly. The EOCs include three different variants: large depression angle variant (EOC1), target configuration variant (EOC2-C), and version variant (EOC2-V). The EOC1 dataset consists of four target classes: 2S1, BRDM2, T72, and ZSU23/4, as shown in Table 2. Images under the depression angle of 17° are used as the training set, while those of 30° are used for the testing. Due to the sensitivity of SAR images to depression angles, it is crucial to evaluate the performance of recognition algorithms using images under different depression angles. In addition, the MSTAR dataset contains multiple target classes, and each class has several serial numbers. Different serial numbers within the same class mainly reflect the difference of the target in local structures. As shown in Table 3, although the test set in the EOC2-C scenario consists of multiple serial numbers, they all belong to the same category, namely T72. Like EOC2-C the training and test sets in EOC2-V are composed of different version numbers in the same class. Further details about the EOC2-V can be found in Table 4.

### 3.2. Experimental Setups

This experiment evaluates the methods proposed in Section 2 based on the MSTAR dataset. First, we use the method in Section 2.1 to segment SAR images to extract the target region and shadow mask as input for subsequent deep networks. In Section 2.1, the iteration number of anisotropic diffusion filtering is set to 20 in Step 2. In Step 5, we use a sliding window of size 5×5 and set the threshold of counting filtering to 15. After experimental validation, it was determined that a distance threshold $T_{D}$ within the range of [40, 50] was suitable. The structural elements employed in the morphological operations in Step 6 are as follows:

(11) $$\left[\begin{array}{ccccc} 0 & 1 & 1 & 1 & 0\\ 1 & 1 & 1 & 1 & 1\\ 1 & 1 & 1 & 1 & 1\\ 1 & 1 & 1 & 1 & 1\\ 0 & 1 & 1 & 1 & 0 \end{array}\right].$$

Then, the data-augmentation method in Section 2.2 is used to segment training set images. Since the FEM proposed in Section 2.3.3 is generalizable, it can be easily embedded into existing SAR image-classification backbones. Therefore, to more comprehensively explore the performance of the proposed FEM, we not only choose classification models with outstanding performance in the SAR image domain, such as A-ConvNets [27], AM-CNN [28], ES-CNN [43], LM-BN-CNN [23], and ESENet [52], but also include some classic models in the optical image domain, such as MobileNetV3 [49] and ResNet [53].

Since different deep networks require different input image sizes, such as the (1, 88, 88) input for A-ConvNets, resizing the input images following the original model’s requirements is essential. Furthermore, the number of input channels for the first convolutional layer in optical image-classification models (MobileNetV3 and ResNet) is changed to 1 to accommodate grayscale SAR images. To adapt to the full azimuth angle imaging of the MSTAR dataset, we applied random center rotations ranging from 0° to 30° to the SAR images in the training set.

We used the Adam optimizer, with an initial learning rate of 0.001, a weight decay of 0.00005 every 20 epochs, and a batch size of 32, and each model was trained for 250 epochs. All deep network models were implemented using the PyTorch framework, with an RTX 2080 Ti GPU and an Intel(R) Xeon(R) Platinum 8255C CPU.

### 3.3. Experimental Results under SOC

In this section, we measure the enhancement effects of the shadow information and FEM under SOC, where the image details of SOC are shown in Table 1. Since each classification model has different inputs, to compare their performance more fairly, we show their recognition accuracy under different input types in Table 5 and Table 6. In this paper, $T_{region}$ and $T_{region}+S_{region}$ represent the target region, the target and shadow regions, respectively. Moreover, $T_{region}$ + $S_{mask}$ represents the target region and shadow mask. The #Params column provides the number of parameters for each model, allowing for comparing their complexity. The bold font in parentheses indicates the additional parameters introduced by embedding FEM. The Accuracy (%) column presents the recognition accuracy of each model under different inputs. Furthermore, comparing the recognition accuracy of ATR models under different operational conditions (i.e., SOC and EOCs) provides a comprehensive assessment of the algorithm’s robustness. The boldface in the Accuracy (%) column represents the accuracy gains achieved by adding FEM or shadow information. It is important to note that when the input is $T_{region}$, it represents the gain of the FEM. However, when the input is $T_{region}+S_{mask}$, it signifies the combined gain of the shadow information and FEM.

As shown in Table 5, when using original SAR images as input, the accuracy of each baseline model reaches more than 99%. However, by taking only the target region as input, the performance of all models will suffer a significant degradation. This degradation indicates that clutter greatly influences extracting depth features [43], which demonstrates the necessity of SAR image segmentation. However, it is worth noting that models incorporating attention mechanisms, such as AM-CNN, ESENet, and MobileNetV3, perform better than the others. Therefore, the performance of baseline models that combine with FEM is improved. For example, A-ConvNets + FEM reaches over 98%, only increasing the parameters by 29 K.

To further investigate the contribution of the shadow to ATR performance, Table 6 presents the recognition results when the input consists of the target and shadow information. If the target and shadow regions are fed directly into the deep model without preprocessing, the accuracy will be lower than using only the target region. Specifically, for ES-CNN, its performance is 96% using the target region; however, its accuracy is just 79% when the input includes both the target and shadow regions. The reason for this is that the intensity of the target is much higher than its shadow, which makes CNN unable to extract shadow features effectively. However, after compensating for shadows using the data augmentation proposed in this paper, the classification accuracy of ES-CNN + FEM reaches 99% when the input includes both the target region and the shadow mask. This indicates that the proposed preprocessing strategy helps CNN to extract discriminative features from the shadows. In addition, the ZSL-Net [55] and Resnet18+IFTS [44] (Table 6) also preprocess the shadow region differently because of considering the unique properties of shadows. To offer a clearer view of the classification performance for each target, Table 7 presents the confusion matrix of A-ConvNets + FEM under SOC when the input is composed of the target region and shadow mask.

It is worth noting that, despite having fewer trainable parameters compared to optical images, the customized CNN models [23,27,28,52] for SAR images still achieve the desired classification performance. Deep networks for optical images often seek more trainable parameters to improve feature representation capabilities. However, this does not apply to SAR images with a limited number of samples. Large deep networks tend to be severely overfitted in SAR images. Therefore, improving SAR ATR performance through deeper backbone models is limited. Considering this problem, this paper primarily explores enhancing CNN performance from the perspective of SAR feature fusion, i.e., the fusion of depth and shadow features. In terms of the number of parameters, on the one hand, the FEM proposed is lightweight enough because of using the DSC. On the other hand, it is embedded in the downsampling layer of the CNN. Consequently, it does not significantly increase the number of parameters of the original deep network, seeing Table 5 and Table 6.

### 3.4. Experimental Results under EOCs

This section investigates the performance enhancement of the shadow mask and FEM under EOCs. Table 8 shows the overall recognition results under EOCs. The experimental results under EOC1, EOC2-C, and EOC2-V are analyzed as follows.

#### 3.4.1. Results under EOC1

As demonstrated in Table 2, EOC1 represents a four-class classification task under a large depression angle variant. As shown in Table 8, even though EOC1 only encompasses four classes compared to SOC, the recognition accuracy of each CNN model declines significantly. For instance, taking only the target region as input, the classification accuracy of existing deep-learning models under SOC generally exceeds 96% (Table 5), while it is around 91% under EOC1 (Table 8). The reason for this is that the target produces more significant distortion under high depression angles. However, all models incorporating FEM achieve performance improvement (gains surpass 1%, see Table 8) compared to the original models. This suggests that FEM contributes to the enhancement of the target depth features. Furthermore, when the input includes target and shadow information, the ATR performance of deep-learning models combined with FEM is further improved, indicating that the shadow can still provide helpful classification features even under large depression angle variations. The confusion matrix of A-ConvNets + FEM under EOC1 is displayed in Table 9.

#### 3.4.2. Results under EOC2-C

As illustrated in Table 3, the training set under EOC2-C comprises BMP2, BRDM2, BTR70, and T72 (132), while the test set consists of T72 targets with different configurations. Like EOC1, when the input is only the target region, the original deep-learning models generally exhibit lower ATR performance (accuracy below 90%), as seen in the EOC2-C column in Table 8. However, existing models combined with FEM achieve varying gains (greater than 1% improvement) using the target information. Furthermore, when the input consists of both the target and shadow, each deep-learning model with FEM achieves its maximum gain. Considering that the test set of EOC2-C has only one class, namely T72, this proves that shadows provide supplementary information about targets under configuration variants. The confusion matrix of A-ConvNets + FEM under EOC2-C is presented in Table 10.

#### 3.4.3. Results under EOC2-V

The EOC2-V reflects different versions of the BMP2 and T72 target types, including four classes for the training set and two classes for the test set (see details in Table 4). As demonstrated in the third column of Table 8, the performance of deep-learning models under EOC2-V is generally lower than that in SOC when the input is just the target region. However, by taking target regions and shadow masks as input, deep models combined with FEM achieve huge performance improvements. Classification accuracy of all models exceeds 97%. Therefore, we can infer that in the case of changes in the local structure of the target (such as the fuel tank), the shadow can still maintain the overall structure of the target. Table 11 shows the confusion matrix of A-ConvNets + FEM under EOC2-V.

### 3.5. Analysis on Results of the SOC and EOCs

According to the experimental results under SOC (Table 5), existing SAR image-classification models have achieved good recognition accuracy when using raw SAR images as input. However, this approach tends to rely on the similarity of background clutter to achieve higher classification scores. Therefore, we can extract the valid and relevant target and shadow features from SAR images for classification by employing image segmentation techniques. This approach avoids the background clutter of SAR images and improves the model’s generalization performance across different scenarios. Notably, we customized the processing of shadows, including intensity and geometric distortion compensation, enabling the CNN model to extract highly discriminative features for classification from the shadow information. Comparing the results in Table 5 and Table 6, the published SAR classification models achieve state-of-the-art performance even when the input only includes target and shadow information after embedding FEM. The experimental results under EOCs (Table 8) further demonstrate the effectiveness of shadow features in extended operational conditions. However, the accuracy of the classification model in EOC1 is lower than in EOC2-C and EOC2-V, mainly due to the more severe shadow distortion caused by large depression angle differences. Therefore, shadow features are unsuitable for cases with significant changes in the depression angles.

It is worth noting that this paper focuses on using CNN to comprehensively extract depth features of targets and shadows to enhance the recognition performance and generalization of existing SAR classification models on the MSTAR dataset. Therefore, for SAR images in complex scenes, modern SAR image segmentation methods can be chosen to ensure the accuracy of shadow extraction [43]. Integrating advanced segmentation methods or classification networks into the proposed SAR target recognition framework will further enhance its universality.

### 3.6. Contributions of the Target and Shadow

In this section, we present a visual analysis of the FEM, which provides some intuitive interpretability of the deep networks and helps us understand the role of FEM in the network. On the one hand, visualizing the spatial attention weights in the FEM helps observe the importance of the target region and shadow mask. On the other hand, we use Grad-CAM to visually analyze the entire FEM, therefore explaining how FEM enhances the performance of existing deep networks. We take the trained A-ConvNets + FEM as an example, with its network structure shown in Figure 9.

**Figure 11:**
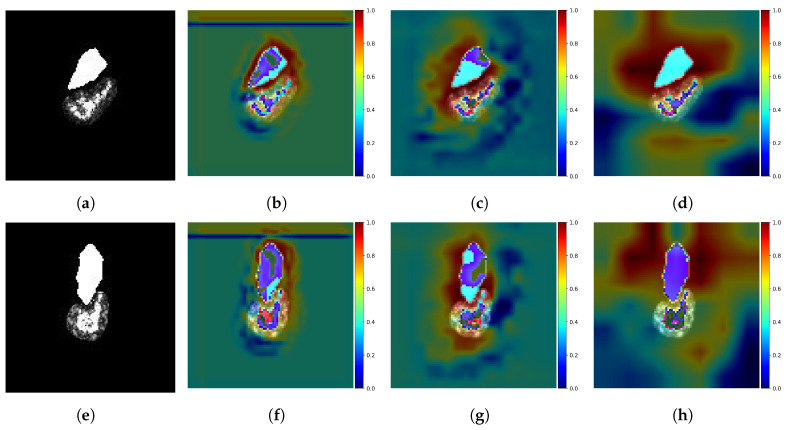
Spatial attention visualization of FEM. (**a**) Target region and shadow mask of T72; (**b**–**d**) are FEM1, FEM2 and FEM3 attention overlays of A-ConvNets + FEM, respectively. (**e**) Target region and shadow of BMP2; (**f**–**h**) are FEM1, FEM2 and FEM3 attention overlays of A-ConvNets + FEM, respectively.

To provide a visualization of the spatial attention weights in the FEM, we overlay the spatial attention weights with the original input image. Specifically, we normalize the attention weight matrix and compute the mean value along the channel dimension. Subsequently, the attention weight matrix is aligned to the input image. Then, a color mapping technique is employed to transform the normalized results into a color heatmap and overlay the heatmap on the input image. Figure 11b–d,f–h illustrate the overlay results of three spatial attention weights in A-ConvNets + FEM combined with the input image. As depicted in Figure 11, high-scattering regions of the target are assigned large weights by FEM because of their rich backscattering characteristic. However, for the shadow, we use their mask as input, allowing deep-learning models to extract depth features from its contour and emphasize the importance of different contour segments. This strategy is consistent with traditional methods that employ shadow contour features for classification [13,14,15]. In addition, Figure 11 shows that the deep models combined with FEM can comprehensively capture the depth features of targets and shadows.

**Figure 12:**
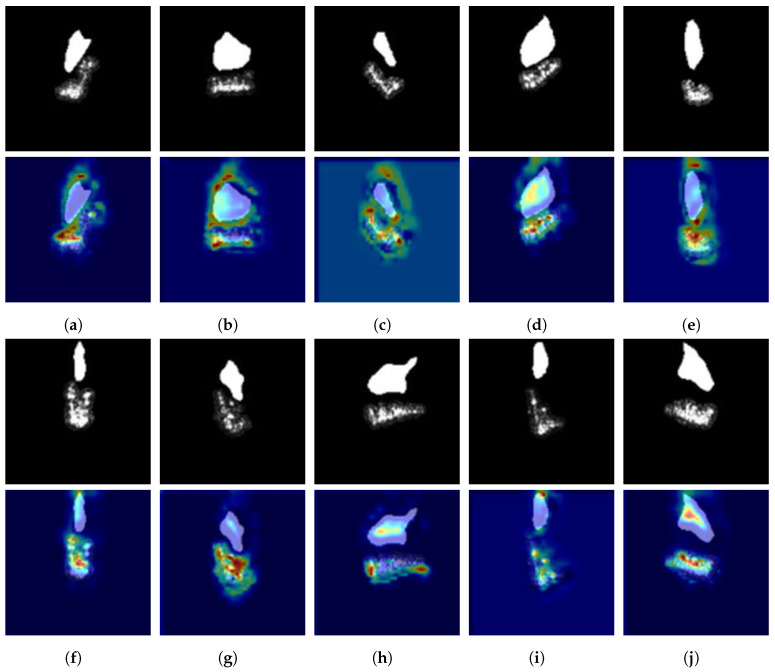
Input images and class activation maps for ten object categories under SOC. (**a**) 2S1. (**b**) BMP2. (**c**) BRDM2. (**d**) BTR60. (**e**) BTR70. (**f**) D7. (**g**) T62. (**h**) T72. (**i**) ZIL131. (**j**) ZSU23/4.

As a visualization technique, Grad-CAM can explain the decision-making process for specific categories in the CNN [56]. It highlights crucial regions in the input image related to the target class, helping us understand the feature-enhancement effect of FEM in deep networks. Figure 12 shows the target area and shadow mask images of ten classes under SOC and their corresponding class activation maps.

**Figure 13:**
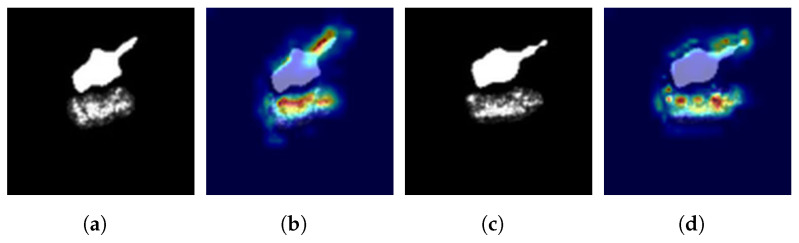
Class activation maps of important parts of T72 (A05). (**a**,**c**) are T72 input images. (**b**,**d**) are their class activation maps, respectively.

From the class activation maps of FEM in Figure 12, when the scattering information of the target area is not clear, FEM will focus on the shadow mask and extract useful depth features for the classifier. Moreover, if the shadow mask provides discriminative features for the classifier, such as the barrel of the T72 (A05) under EOC2-V, FEM will generate a large amount of activation around the gun barrel in the class activation map, as shown in Figure 13.

**Figure 14:**
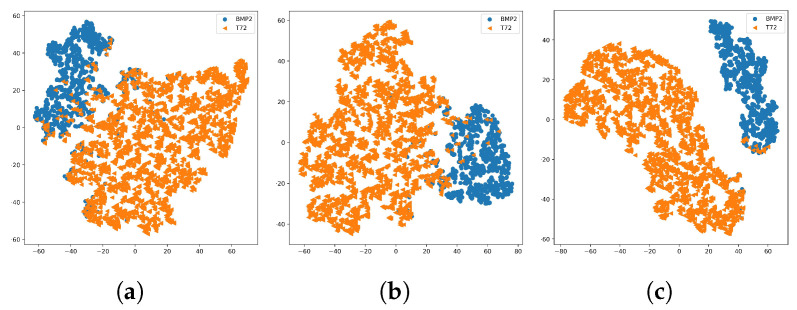
t-SNE visualization of output features of A-ConvNets + FEM under EOC2-V. (**a**,**b**) are the output features of A-ConvNets and A-ConvNets + FEM under only the target region, respectively. (**c**) The output features of A-ConvNets + FEM under the target region and shadow mask.

To intuitively demonstrate the effectiveness of shadows from a global perspective, we visualized the high-dimensional feature vectors extracted by different models using the t-SNE algorithm [57]. Figure 14 displays the distribution of high-dimensional features obtained by A-ConvNets and A-ConvNets + FEM under various input conditions. Because the test set of EOC2-V only includes two main target types, each color in the figure represents a different target type. It can be observed that, compared to the high-dimensional features obtained solely from the target region (Figure 14a,b), the high-dimensional features acquired by A-ConvNets + FEM from both the target region and the shadow mask exhibit enhanced separability. This qualitatively confirms that shadow features can still provide effective classification characteristics under variant configuration scenarios.

## 4. Conclusions

Shadows in SAR images can reveal the structural information of the target from a side perspective, providing unique features distinct from the target itself. However, shadows exhibit properties of low intensity and depression angle sensitivity, which make it challenging for CNN to extract useful information from them. To address this problem, we propose a novel strategy for fusing target and shadow information to enable CNN to extract depth features from targets and shadows comprehensively. First, we introduce a segmentation method to extract the target and shadow information. Taking the target region and shadow mask as input to CNN helps solve the shadow’s low-amplitude issue, enabling subsequent networks to extract deep representation from the shadow contour. Second, we propose a data-augmentation technique to compensate for the geometric distortion of shadows due to different depression angles. Finally, we present a FEM that can adaptively fuse the target and shadow information while emphasizing the partial importance of targets and shadows. Extensive experiments conducted on the MSTAR dataset demonstrate that the FEM can improve the ability of existing networks to extract information on target and shadow, therefore achieving state-of-the-art performance in both SOC and EOC scenarios.

Future work includes the following aspects. First, advanced segmentation methods, such as deep-learning-based SAR image segmentation, can be utilized to improve target and shadow extraction in complex scenes. Second, the proposed FEM can be integrated into deep backbone networks to enhance recognition accuracy; however, this may increase the complexity of the models. Lastly, integrating the proposed method with the modern SAR ATR framework can help in handling SAR images with multiple targets. 

## Figures and Tables

**Table 1:** Detailed Information of Targets Under SOC.

Class	SerNum	Training		Test	
		Depression	Number	Depression	Number
2S1	B01	$17^{\circ }$	299	$15^{\circ }$	274
BMP2	9566	$17^{\circ }$	232	$15^{\circ }$	195
BRDM2	E-71	$17^{\circ }$	298	$15^{\circ }$	274
BTR60	7532	$17^{\circ }$	256	$15^{\circ }$	195
BTR70	c71	$17^{\circ }$	233	$15^{\circ }$	196
D7	13015	$17^{\circ }$	299	$15^{\circ }$	274
T62	A51	$17^{\circ }$	299	$15^{\circ }$	273
T72	132	$17^{\circ }$	232	$15^{\circ }$	196
ZIL131	E12	$17^{\circ }$	299	$15^{\circ }$	274
ZSU23/4	d08	$17^{\circ }$	299	$15^{\circ }$	274

**Table 2:** Detailed Information of Targets Under EOC1.

Class	SerNum	Training		Test	
		Depression	Number	Depression	Number
2S1	B01	$17^{\circ }$	299	$30^{\circ }$	288
BRDM2	E-71	$17^{\circ }$	298	$30^{\circ }$	287
T72	A64	$17^{\circ }$	299	$30^{\circ }$	288
ZSU23/4	d08	$17^{\circ }$	299	$30^{\circ }$	288

**Table 3:** Detailed Information of Targets Under EOC2-C.

Class	SerNum	Training		Test	
		Depression	Number	Depression	Number
BMP2	9563	$17^{\circ }$	233	-	-
BRDM2	E-71	$17^{\circ }$	298	-	-
BTR70	c71	$17^{\circ }$	233	-	-
T72	132	$17^{\circ }$	232	-	-
	A32	-	-	$17^{\circ },15^{\circ }$	572
	A62	-	-	$17^{\circ },15^{\circ }$	573
	A62	-	-	$17^{\circ },15^{\circ }$	573
	A64	-	-	$17^{\circ },15^{\circ }$	573
	S7	-	-	$17^{\circ },15^{\circ }$	419

**Table 4:** Detailed Information of Targets Under EOC2-V.

Class	SerNum	Training		Test	
		Depression	Number	Depression	Number
BMP2	9563	$17^{\circ }$	233	-	-
	9566	-	-	$17^{\circ },15^{\circ }$	428
	C21	-	-	$17^{\circ },15^{\circ }$	429
BRDM2	E-71	$17^{\circ }$	298	-	-
BTR70	C71	$17^{\circ }$	233	-	-
T72	132	$17^{\circ }$	232	-	-
	812	-	-	$17^{\circ },15^{\circ }$	426
	A04	-	-	$17^{\circ },15^{\circ }$	573
	A05	-	-	$17^{\circ },15^{\circ }$	573
	A07	-	-	$17^{\circ },15^{\circ }$	573
	A10	-	-	$17^{\circ },15^{\circ }$	567

**Table 5:** Accuracy of different methods based on original image and target information under SOC.

Input	Model	#Params	Accuracy (%)
Original Image	A-ConvNets [27]	303 K	99.13
	AM-CNN [28]	2.5 M	99.35
	CA-Net [54]	0.7 M	99.59
	MVGGNet [37]	16.8 M	99.27
	DS-AE-Net [30]	11.2 M	99.30
$T_{region}$	A-ConvNets [27]	303 K	95.12
	AM-CNN [28]	2.5 M	97.59
	ES-Net [43]	95 K	96.41
	LM-BN-CNN [23]	141 K	96.44
	ESENet [52]	551 K	97.32
	ResNet18 [53]	11.2 M	96.57
	MobileNetV3 [49]	2.6 M	97.60
$T_{region}$	A-ConvNets + FEM	332 K (+**29 K**)	98.47 (+**3.35**)
	AM-CNN + FEM	3.0 M (+**0.5 M**)	98.68 (+**1.12**)
	ES-Net + FEM	168 K (+**73 K**)	96.98 (+**0.57**)
	LM-BN-CNN + FEM	187 K (+**46 K**)	98.14 (+**1.70**)
	ESENet + FEM	597 K (+**46 K**)	98.43 (+**1.11**)
	MobileNetV3 + FEM	2.6 M	98.13 (+**0.53**)

**Table 6:** Accuracy of different methods based on target and shadow information under SOC.

Input	Model	#Params	Accuracy (%)
$T_{region}$ + $S_{region}$	ES-CNN [43]	95 K	79.10
	ZSL-Net [55]	-	91.93
	ResNet18 + IFTS [44]	22 M	98.90
$T_{region}$ + $S_{mask}$	A-ConvNets + FEM	332 K (+**29 K**)	99.71 (+**4.59**)
	AM-CNN + FEM	3.0 M (+**0.5 M**)	99.75 (+**2.16**)
	ES-Net + FEM	168 K (+**73 K**)	99.34 (+**2.93**)
	LM-BN-CNN + FEM	187 K (+**46 K**)	99.46 (+**3.02**)
	ESENet + FEM	597 K (+**46 K**)	99.58 (+**2.26**)
	MobileNetV3 + FEM	2.6 M	99.71 (+**2.11**)

**Table 7:** Confusion matrix of the A-ConvNets + FEM under SOC.

Class	2S1	BMP2	BRDM2	BTR60	BTR70	D7	T62	T72	ZIL131	ZSU23/4	Acc (%)
2S1	266	0	0	0	1	0	0	0	0	0	99.62
BMP2	0	195	0	0	1	0	0	0	0	0	99.48
BRDM2	0	0	274	0	0	0	0	0	0	0	100
BTR60	0	0	1	189	0	0	0	0	0	0	99.47
BTR70	0	0	1	0	195	0	0	0	0	0	99.48
D7	1	0	0	0	0	269	0	0	1	2	98.53
T62	0	0	0	1	0	1	269	1	0	1	98.53
T72	0	0	0	0	0	0	0	196	0	0	100
ZIL131	0	0	0	0	0	0	0	0	274	0	100
ZSU23/4	0	0	0	0	0	0	0	0	0	269	100
Average											99.50

**Table 8:** Accuracy of different methods based on target and shadow information under EOCs.

Input	Model	#Params	Accuracy(%)		
			EOC1	EOC2-C	EOC2-V
$T_{region}$	A-ConvNets [27]	303 K	92.13	88.74	88.26
	AM-CNN [28]	2.5 M	92.35	89.51	92.10
	ES-CNN [43]	95 K	90.74	87.96	86.85
	LM-BN-CNN [23]	141 K	91.90	90.05	88.60
	ESENet [52]	551 K	92.93	89.58	89.35
	Resnet18 [53]	11.2 M	91.79	90.10	92.50
	MobileNetV3 [49]	2.6 M	91.92	90.93	91.45
	A-ConvNets + FEM	332 K (+**29 K**)	93.18 (+**0.97**)	89.98 (+**1.24**)	92.08 (+**5.00**)
	AM-CNN + FEM	3.0 M (+**0.5 M**)	94.26 (+**1.91**)	90.13 (+**0.62**)	93.84 (+**1.74**)
	LM-BN-CNN + FEM	187 K (+**46 K**)	93.74 (+**1.84**)	91.22 (+**1.17**)	92.13 (+**4.13**)
	ESENet + FEM	597 K (+**46 K**)	94.45 (+**1.52**)	91.58 (+**2.00**)	93.42 (+**4.07**)
	MobileNetV3 + FEM	2.6 M	94.00 (+**2.08**)	92.33 (+**1.40**)	92.95 (+**1.05**)
$T_{region}$ + $S_{mask}$	A-ConvNets + FEM	332 K (+**29 K**)	96.13 (+**4.00**)	97.56 (+**8.82**)	98.09 (+**9.83**)
	AM-CNN + FEM	3.0 M (+**0.5 M**)	96.60 (+**2.34**)	98.10 (+**8.59**)	98.15 (+**6.05**)
	LM-BN-CNN + FEM	187 K (+**46 K**)	96.87 (+**4.97**)	97.20 (+**7.05**)	97.91 (+**9.31**)
	ESENet + FEM	597 K (+**46 K**)	97.39 (+**4.46**)	97.71 (+**8.13**)	98.22 (+**8.87**)
	MobileNetV3 + FEM	2.6 M	95.48 (+**3.56**)	98.53 (+**7.60**)	97.67 (+**6.22**)

**Table 9:** Confusion matrix of the A-ConvNets + FEM under EOC1.

Class	2S1	BRDM2	T72	ZSU23/4	Acc (%)
2S1	277	7	3	1	96.18
BRDM2	0	287	0	0	100
T72	5	6	264	13	91.66
ZSU23/4	0	0	9	269	93.40
Average					95.31

**Table 10:** Confusion matrix of the A-ConvNets + FEM under EOC2-C.

Class	SerNum	BMP2	BRDM2	BTR70	T72	Acc (%)
T72	A32	9	3	0	560	97.90
	A62	8	0	1	564	98.43
	A63	9	0	0	564	98.43
	A64	22	0	2	549	95.81
	S7	10	1	1	407	97.13
Average						97.54

**Table 11:** Confusion matrix of the A-ConvNets + FEM under EOC2-V.

Class	SerNum	BMP2	BRDM2	BTR70	T72	Acc (%)
BMP2	9566	406	0	0	22	94.86
	C21	389	1	2	37	90.67
T72	812	6	0	0	420	98.59
	A04	0	0	0	573	100
	A05	0	0	0	573	100
	A07	0	0	0	573	100
	A10	0	0	0	567	100
Average						97.73

## Data Availability

Not applicable.
